# Coated polymeric needles for rapid and deep intradermal delivery

**DOI:** 10.1016/j.ijpx.2020.100048

**Published:** 2020-05-04

**Authors:** Álvaro Cárcamo-Martínez, Qonita Kurnia Anjani, Andi Dian Permana, Ana Sara Cordeiro, Eneko Larrañeta, Ryan F. Donnelly

**Affiliations:** a School of Pharmacy, Queen's University Belfast, Medical Biology Centre, 97 Lisburn Road, Belfast BT9 7BL, UK; b Department of Pharmaceutics, Faculty of Pharmacy, Hasanuddin University, Makassar, Indonesia

**Keywords:** Polymeric needles, Intradermal delivery, Rhodamine B, Optical coherence tomography

## Abstract

Two groups of single polymeric needles (crosslinked Gantrez®S-97 and poly(ethylene glycol)) of different lengths (2 mm and 4.5 mm) with defined base widths were fabricated and tested in terms of their mechanical strength and insertion abilities using two skin models (Parafilm® and porcine skin). For the shorter needles, application of an axial force (32 N) resulted in a height reduction of approximately 80%. Nonetheless, around 80% of total needle length was successfully inserted in both skin models. Optical coherence tomography showed that base width highly impacted insertion capabilities of the longer needles as only the thicker one (0.922 mm width at base) inserted into porcine skin. Additionally, needles were coated with rhodamine B and inserted into porcine skin. In comparison to a control, penetration depth of the model drug increased 2-fold for short and 4.5-fold for long needles, respectively. Moreover, quantification across skin sections showed that shorter needles delivered 10 μg of the compound in a depth of 1.5–2.0 mm while long needles were capable of delivering 5 μg into even deeper skin layers (2.0–3.0 mm), confirming the potential of coated polymeric needles for rapid and deep intradermal delivery.

## Graphical abstract

**Figure:**
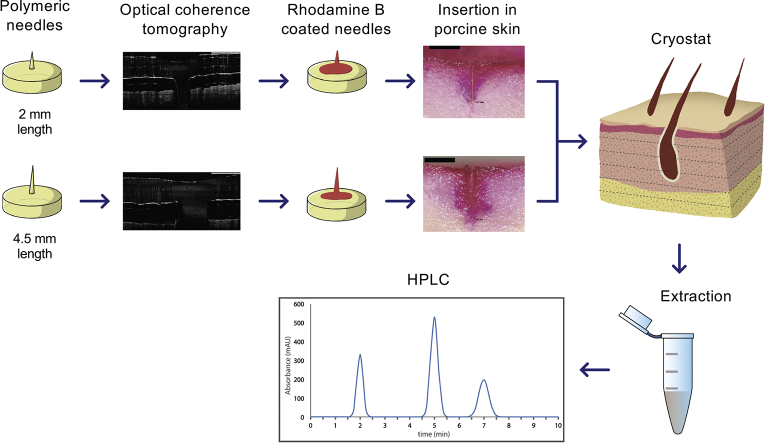

## 1 Introduction

The oral route is currently the most common path for drug administration because of its simplicity and non-invasive character. However, metabolic activities in the gastrointestinal tract, including intestinal and hepatic metabolism, may decrease the bioavailability of drugs delivered *via* this route (Pereira De Sousa and Bernkop-Schnürch, 2014). Hence, higher drug doses need to be used, increasing the probability of side effects and drug interactions. In this sense, alternative drug delivery pathways could represent a better option.

Skin is the most accessible and largest organ of the body with a surface area of 1.7 m^2^ (Benson and Watkinson, 2011). The *stratum corneum* is the outermost sublayer of the epidermis and is composed, *inter alia*, by corneocytes (protein-enriched cells) embedded in an extracellular lipid mixture of ceramides, cholesterol, and free fatty acids. Both corneocytes and this lipid mixture serve as barrier against the percutaneous penetration of chemicals and microbes (Benson and Watkinson, 2011; Menon et al., 2012; Proksch et al., 2008), limiting diffusion of drugs into the skin. To circumvent this barrier and exploit the potential of skin for intra and transdermal drug delivery, advanced methodologies such as nanocarriers (liposomes, solid lipid nanoparticles, polymer nanoparticles), sonophoresis, iontophoresis and microneedle arrays (MNs), among others have been reported (Azagury et al., 2014; Iqbal et al., 2018; Kennedy et al., 2017; Larrañeta et al., 2016a; Watkinson et al., 2016). MNs have received great attention due to their capacity to overcome the *stratum corneum* barrier by piercing it and deliver drugs into the epidermis and dermal microcirculation, bypassing disadvantages of conventional hypodermic needles such as needle-stick injuries and generation of hazardous waste (Larrañeta et al., 2016a; Rzhevskiy et al., 2018). Interestingly, MNs can be fabricated from mono, di and polysaccharides and biocompatible polymers loaded with the drug, using moulding techniques. After insertion, polymers will be in contact with the interstitial fluid and dissolution or swelling takes place, releasing the drug into the epidermis or upper dermis (Donnelly et al., 2012; Ramöller et al., 2019). Since these processes may take time, inclusion of drugs on the surface of MNs using coating formulations has been investigated in order to speed up the release. Dip coating, gas-jet drying, spray drying, ink-jet printing are some of the techniques reported (Haj-Ahmad et al., 2015; Ingrole and Gill, 2019; Tarbox et al., 2018), which have been tested for the development of coated MNs with influenza vaccine, lidocaine, insulin, miconazole, 5-fluorouracil, among others (Baek et al., 2017; Boehm et al., 2014; Choi et al., 2012; Ross et al., 2015; Uddin et al., 2015).

Despite rapid drug dissolution associated with coated MNs, diffusion of drug into the skin takes longer and so MNs of increased length could represent a better alternative when the aim is to reach the dermis. For this purpose, intradermal microinjection systems have been fabricated using single hollow needles. Gupta et al. reported a study on fluid injection mechanics using borosilicate MNs with lengths between 500 μm and 4 mm (Gupta et al., 2011b). Subsequently, subcutaneous catheters and 900 μm length borosilicate MNs were compared for insulin delivery, and reported that intradermal infusion with MNs led to an insulin peak in half the time of catheters (Gupta et al., 2011a). This type of technology reached the market in 2009 when Sanofi Pasteur obtained a marketing authorisation by the European Medicine Agency (EMA) for Intanza®, an inactivated influenza vaccine. Using a microinjection system composed of a single hollow metallic needle of 1.5 mm length and a solution containing three different strains of influenza virus, the vaccine is delivered intradermally (European Medicines Agency, 2018).

In the present work, a study combining the use of polymeric single needles coated with a model drug, rhodamine B, for intradermal delivery is described. Polymers offer the advantage of enhanced biocompatibility when compared to silicon, glass and ceramics MNs as well as metal hollow microneedles by circumventing the risk of fracture and fragments being left on the skin (Larrañeta et al., 2016a; McConville et al., 2018; O'Mahony, 2014; Yang et al., 2019). Two groups of polymeric needles of defined lengths and widths at base were manufactured, testing the effect of both factors on mechanical strength and insertion properties. Then, needles were coated with rhodamine B to investigate how deep they can deliver a cargo in a skin model, quantifying it as well across skin sections and evaluating the potential of this approach for rapid intradermal drug delivery.

## 2 Materials

Hypodermic needles U-100 were purchased from Beckton Dickinson (Cedex, France) and metallic needles, ranging between 0.470 and 1.2 mm in base width (measured at 5 mm from the tip) were purchased from Korbond Industries Ltda (Lincolnshire, UK). Loctite®, cyanoacrilate glue, was purchased from Henkel (Hertfordshire, England). Gantrez®S-97, a *co*-polymer of methylvinylether and maleic acid (PMVE/MA, molecular weight 1,500,000 Da) and Plasdone™ K29/32, poly(vinyl)pyrrolidone (PVP), were provided by Ashland (Kidderminster, UK). Glycerol bidistilled 99.5% was obtained from VWR (Leicestershire, UK). Parafilm M® was purchased from Brand GmbH (Wertheim, Germany). Poly(caprolactone), Capa™ 6800 (molecular weight 80,000 Da) was obtained from Perstorp (Malmö, Sweden). Poly(urethane) needle testing foil Deka® was kindly provided by Melab GmbH (Leonberg, Germany). Poly(lactic) acid (PLA), molecular weight 60,000 was purchased from Ultimaker B.V. (Geldermalsen, The Netherlands). Poly(ethyleneglycol) molecular weight 200 Da (PEG), rhodamine B, Tween® 80 (poly(ethylene glycol) sorbitan monooleate), were purchased from Sigma-Aldrich (Steinheim, Germany). Silastic® S, a silicone rubber and curing agent mix, was purchased from Thompson Bros. Ltd. (Newcastle Upon Tyne, UK). Excised porcine skin with underlying connective tissue (around 3.5 cm in depth) was obtained from a local butcher and immediately frozen at −20 °C until use.

## 3 Methods

### 3.1 Manufacture of master moulds, silicone templates and single hydrogel-forming needles

**Fig. 1:**
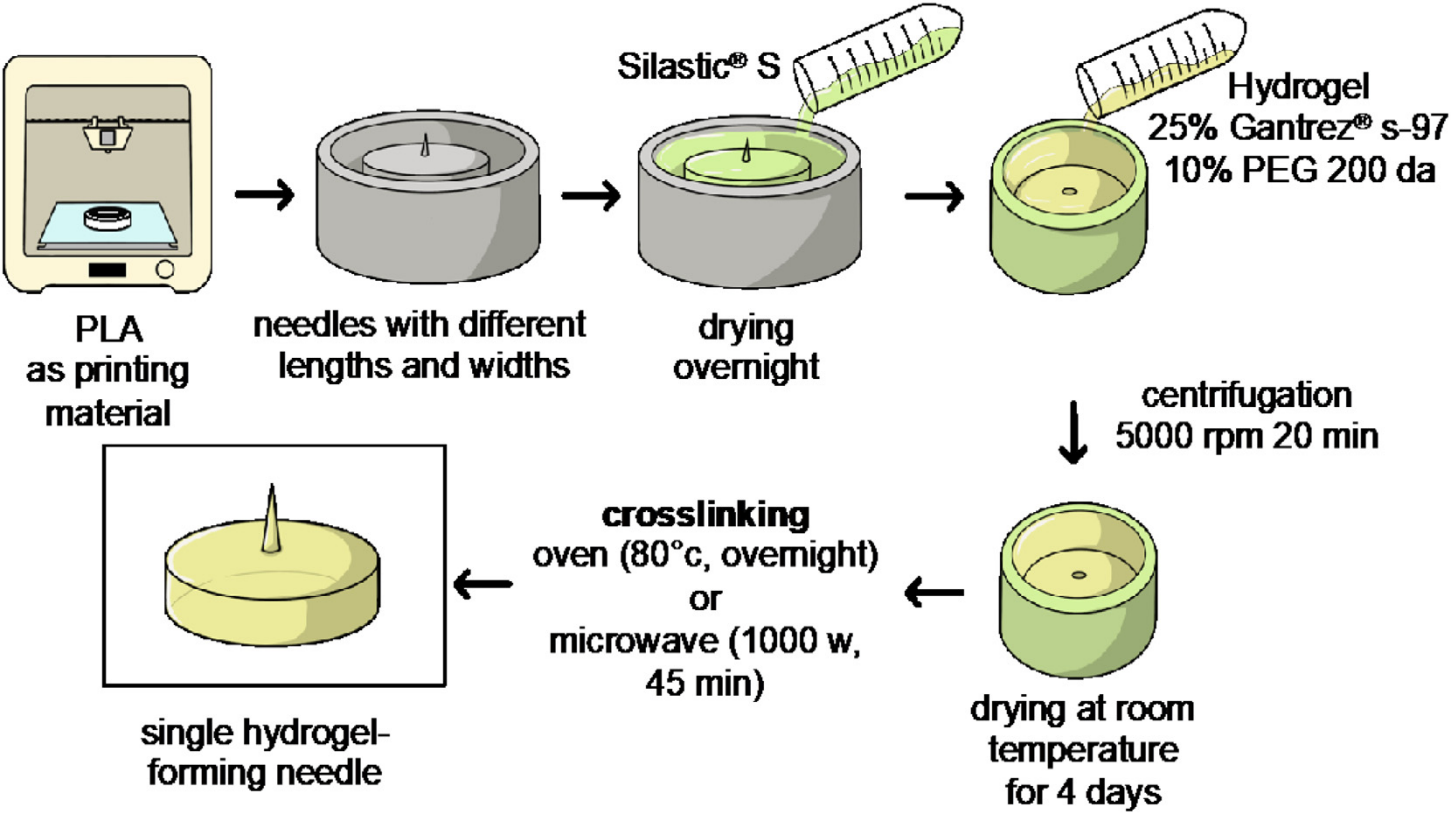
Fabrication of master moulds, silicone moulds and single hydrogel-forming needles.

Master moulds were manufactured with a 3D printer Ultimaker 3 (Ultimaker B.V., Geldermalsen, The Netherlands) using PLA as printing material for the base. Then, hypodermic or metallic needles with increasing widths at base and different lengths were inserted and glued to these bases using cyanoacrilate. A mixture of Silastic® S and a curing agent, in a proportion of 10:1, was poured into the master moulds and allowed to cure overnight to obtain silicone moulds. Afterwards, single hydrogel-forming needles were prepared by pouring an aqueous mixture of 25% w/w Gantrez® S-97 and 10% w/w PEG 200 Da into the silicone moulds, followed by centrifugation at 5000 rpm for 20 min. The mixture was allowed to dry in the moulds at room temperature for 4 days and the formed needles were removed from the moulds and crosslinked, testing two methods: using either a conventional household Panasonic NN-CF778S microwave (Panasonic UK Ltd., Bracknell, UK) for 45 min at 1000 W or by placing them in a convection oven at 80 °C overnight (Larrañeta et al., 2015). Schematic representation for fabrication of master moulds and hydrogel-forming needles is shown in Fig. 1. Prepared needles were visually inspected to assess their dimensions using a light microscope Leica EZ4 D stereo (Leica Microsystems, Milton Keynes, UK).

### 3.2 Evaluation of the mechanical properties of needles

Mechanical strength of needles was tested with a Texture Analyser TA-XT2 (Stable Microsystems, Haslemere, UK) set in compression mode (pre-test speed: 1 mm/s, test speed: 1 mm/s and post-test speed: 1 mm/s) and using a force of 32 N per 30 s as the target mode (González-Vázquez et al., 2017). Needles were attached with double-side tape to the mobile probe and compressed against a flat metallic surface on the bottom of the equipment. The length of the needles before and after compression was recorded using light microscopy and the percentage of height reduction was calculated.

### 3.3 Needles penetration into Deka® film

**Fig. 2:**
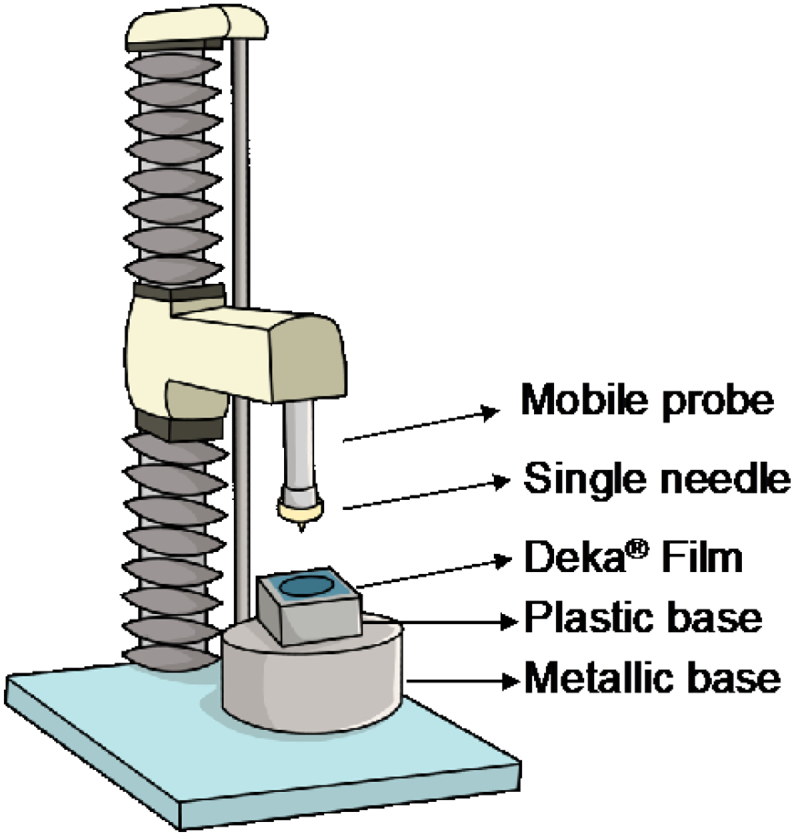
Schematic representation of the Texture Analyser setup for Deka® film penetration studies.

Single hydrogel-forming needles were tested for their ability to pierce Deka® film (0.08 mm and 0.4 mm in thickness), commonly used for the assessment of hypodermic needles (Deutsches Institut für Normung, 2009). As it is shown in Fig. 2, a piece of film was cut and attached with adhesive tape into a plastic base and needles were placed on the mobile probe of the Texture Analyser with double-sided tape. The equipment was set in compression mode (pre-test speed: 1 mm/s, test speed: 2 mm/s and post-test speed: 1 mm/s) and the target mode was distance, selecting the length of each needle tested. Optical coherence tomography (OCT), EX1201 OCT Microscope (Michelson Diagnostics Ltd., Kent, UK), was used to visualise films and confirm that needles were able to pierce them.

### 3.4 Insertion studies in skin models

To test the insertion depth of needles in skin models, Parafilm® and porcine skin were used (Larrañeta et al., 2014). In both cases, needles were attached to the mobile probe of the Texture Analyser and the skin model was placed on the metallic base. The equipment was set in compression mode (pre-test speed: 1 mm/s, test speed: 1 mm/s and post-test speed: 1 mm/s), the target mode was force (32 N) and the test was run for 30 s.

#### 3.4.1 Parafilm® insertion

The Parafilm insertion model developed by Larrañeta et al. was used (Larrañeta et al., 2016b, Larrañeta et al., 2014). Consecutive layers of Parafilm® (20 layers for short needles, equivalent to 2.54 mm in thickness or 40 layers for long needles, equivalent to 5.08 mm in thickness) were used. After the study, each Parafilm® layer was observed with light microscopy, using the last layer pierced to calculate the percentage of the needle's height effectively inserted.

#### 3.4.2 Porcine skin insertion

Skin was defrosted at room temperature and kept in phosphate buffer saline pH 7.4 (PBS) for at least 30 min before use. Once needles were inserted, OCT was used to measure the percentage of needle's height effectively inserted, making a correlation between pixels and μm (1 pixel equals to 4.2 μm) (Larrañeta et al., 2014).

### 3.5 Coating of single needles with rhodamine B

A mixture (% w/w) of 2% rhodamine B, 20% PVP, ethanol 99.5% (q.s. 5 g) and different ratios of glycerol and Tween® 80, as described in Table 1, were investigated for the coating of single-hydrogel forming needles.

**Table 1:** Formulations investigated for the coating of single hydrogel-forming needles.

Excipients	F1	F2	F3	F4	F5
Glycerol	15%	10%	5%	5%	2.5%
Tween® 80	10%	10%	5%	2.5%	2.5%

Blends were sonicated for 30 min to remove any air bubbles. Then, 500 μL of each mixture were cast on to the siliconised surface of a release liner and left to dry at room temperature to evaluate the film-forming capacity of each blend. For the coating, 10 μL of coating formulation were dropped on top of the needles and left to dry at room temperature.

### 3.6 Scanning electron microscopy (SEM)

Needles were observed with a Tabletop TM3030 scanning electron microscope (Hitachi, Tokyo, Japan) to evaluate the coating distribution and determine whether a homogenous coating film was formed. Needles were attached to a double-side carbon conductive paper and placed in an aluminium sample holder. Equipment was set in a low vacuum mode at a voltage of 15 kV.

### 3.7 Rhodamine B penetration depth and quantification in porcine skin sections

Coated needles were inserted manually into porcine skin by pressing for 30 s. After that, a metallic cylindrical weight (15 g) was placed on top of the needles to avoid them being pushed out of the skin and left for 15 min. After removal, penetration depth of rhodamine B was assessed by cutting skin samples with a scalpel and visualizing them with light microscopy. Quantification of rhodamine B across the skin was evaluated by sectioning samples in 50 μm thickness using a Leica CM1900 Cryostat (Leica Microsystems, Nussloch, Germany) and collecting 10 consecutive slices into Eppendorf tubes. To analyse the amount of drug distribution across the skin, 1 mL of methanol was added to the tubes and samples were homogenised with a Tissue Lyser LT (Qiagen, Ltd., Manchester, UK) at 50 Hz for 15 min to extract the drug from the skin (Permana et al., 2019). Then, samples were centrifuged at 14,000 ×*g* for 15 min. An aliquot of supernatant was collected and analysed using reversed-phase HPLC. An Agilent Technologies 1220 Infinity compacted LC Series consisting of Agilent degasser, binary pump, auto standard injector and fluorescence detector (λex 550 nm, λem 580 nm and a gain of 10) (Agilent Technologies UK Ltd., Stockport, UK) was used. Phase separation was performed on a Symmetry® C4 column (50 mm × 4.6 mm with 5 μm particle size) at room temperature. The mobile phase consisted of purified water and an organic mixture (80% methanol and 20% acetonitrile) in a proportion 47:53 v/v. The injection volume and flow rate were 10 μL and 1 mL/min, respectively. Chromatograms were analysed using Agilent ChemStation® Software B.02.01. The analytical method was validated as per the International Committee of Harmonization (ICH) 2005. Table 2 shows the slope, y-inter- cept and coefficient of determination (R2 value) obtained from least squares linear regression analysis, followed by correlation analysis of the calibration plots for rhodamine B.

**Table 2:** Calibration curve properties for quantification of rhodamine B in methanol.

Slope	y-intercept	Linear range (μg/mL)	R^2^	LoD (μg/mL)	LoQ (μg/mL)
712.91	−17.838	0.156–5	0.99997	0.040	0.122

### 3.8 Statistical analysis

Data are shown as mean ± standard deviation (SD) from triplicate measurements, unless otherwise stated. Differences between study groups were assessed for significance using one-way analysis of variance (ANOVA), followed by a multiple comparisons test (Tukey's test) or with a *t*-test (for 2 groups). In both cases, the threshold for significance was *p* < .05. Statistical analysis was performed using GraphPad Prism® version 7 (GraphPad Software, San Diego, CA, USA).

## 4 Results and discussion

### 4.1 Manufacture of master moulds, silicone templates and single hydrogel-forming needles

**Fig. 3:**
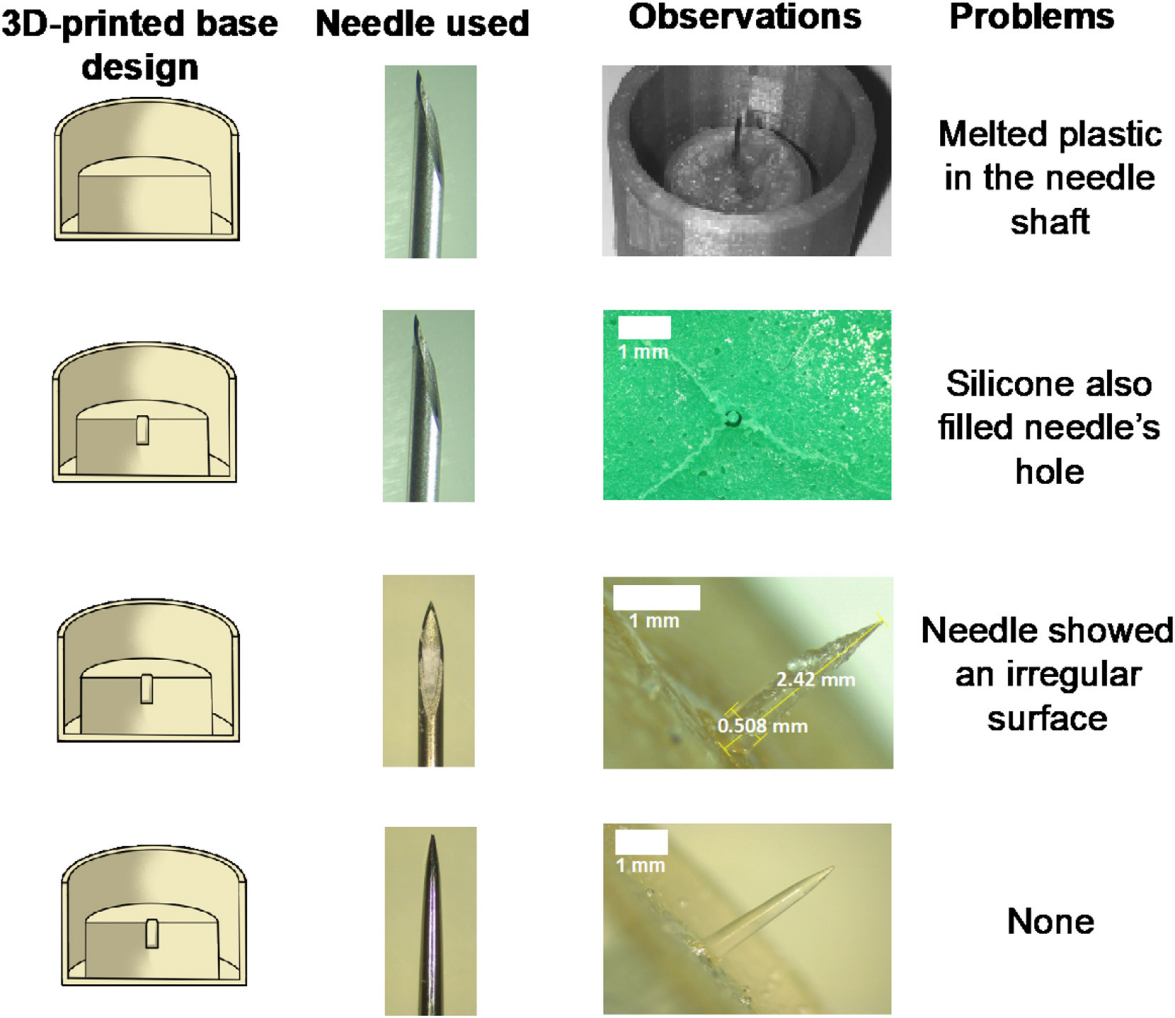
Master and silicone mould prototypes: A) Hypodermic needle glued using a syringe's plunger flange; B) 3D-printed mould including a hole for hypodermic needle insertion; C) Hypodermic needle filled with poly(caprolactone); D) Metallic needles (scale bar represents 1 mm).

Several approaches were followed for the fabrication of master templates. Fig. 3 shows the different prototypes developed and the main issues encountered.

Several issues were faced during the preparation of moulds. Initially, needles were attached to a 3D-printed base using a syringe's plunger flange. Since the needle was attached to the flange by heating it and melting the poly(propylene) (Fig. 3A), plastic got attached to the needle, making it a non-ideal process of fabrication for master moulds as the needles would not be reproducible. In a second attempt (Fig. 3B), a hole was added to the 3D design, which made possible to fix the needle to the printed template using glue. However, it was noticed at this point that the silicone mould prepared using this template had a flaw, since the silicone mixture filled also hypodermic needle's internal hole. To overcome this issue, (Fig. 3C), hypodermic needles were filled with melted PCL before pouring the silicone mixture. Unfortunately, hydrogel-forming needles obtained by this process showed an irregular surface as the result of an uneven distribution of the hydrogel throughout the needle tip. Therefore, metallic needles were used as template instead of hypodermic ones (Fig. 3D). These needles were cut and glued into the moulds and silicone templates were further prepared. Needles obtained with these moulds were crosslinked in an oven at 80 °C overnight or in a conventional household microwave for 45 min at 1000 W. Since needles were crosslinked in the oven inside the silicone moulds, it is quite likely that the water did not adequately leave the polymer mixture, leading to needles with air bubbles within the needle shaft. On the other hand, needles crosslinked in the microwave were removed from the moulds before crosslinking to avoid damaging the silicone mould. These needles were crosslinked in less time and had a smooth surface without air bubbles. Therefore, microwave crosslinking was used for further experiments.

### 4.2 Physical evaluation of single hydrogel-forming needles

**Fig. 4:**
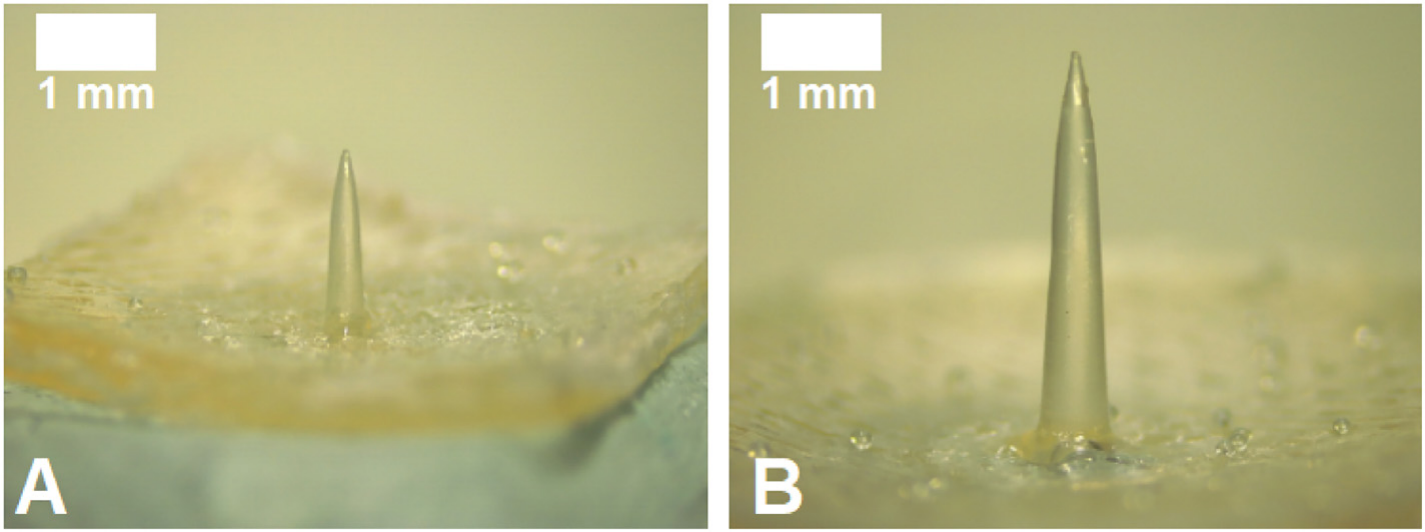
Representative images of single hydrogel-forming needles. A) 2 mm needle, prototype 1A; B) 4.5 mm needle, prototype 2D (scale bar represent 0.5 mm for A and 1 mm for B).

Hydrogel-forming needles obtained were evaluated in terms of length and base width using light microscopy. Representative images of these prototypes are shown in Fig. 4 and dimensions are shown in Table 3.

**Table 3:** Physical properties of single hydrogel-forming needles (mean ± SD, *n* = 5).

Prototype	Length (mm)	Base width (mm)
1A	2.11 ± 0.03	0.464 ± 0.003
1B	2.02 ± 0.04	0.516 ± 0.012
1C	2.09 ± 0.07	0.681 ± 0.004
1D	2.26 ± 0.01	0.890 ± 0.007
2A	4.41 ± 0.10	0.567 ± 0.0012
2B	4.35 ± 0.10	0.638 ± 0.030
2C	4.35 ± 0.07	0.853 ± 0.008
2D	4.51 ± 0.11	0.922 ± 0.060

Two groups of needles were successfully manufactured in a reproducible manner: shorter ones approximately 2 mm in length and longer ones, approximately 4.5 mm in length. For each group, needles with increasing and defined base widths were prepared to evaluate the effect of this parameter on their mechanical and insertion properties and then, compare if the use of either short or long needles leads to a deeper delivery of a model drug into a skin model.

### 4.3 Mechanical properties of single-hydrogel forming needles

**Fig. 5:**
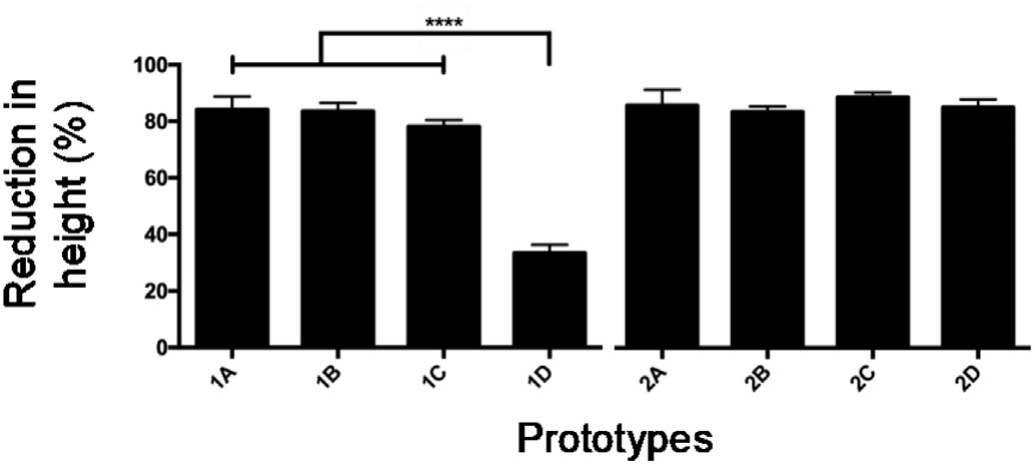
Mechanical properties for both groups of needles (mean + SD, *n* = 3) (****p < .0001).

Single needles were assessed with a Texture Analyser to evaluate their resistance to being pressured against a surface, using an axial force. Fig. 5 shows the percentage of height reduction for each prototype.

Previous studies have shown that the mean force applied by a person on a MNs patch was around 32 N (Larrañeta et al., 2014; Lutton et al., 2015), hence, this was the value used for all the studies. Both types of needles experienced a reduction of around 80% of their height, except for prototype 1D, which showed to be more resistant to compression. Therefore, one way of preventing their compression is to increase base width, increasing their cross sectional area and improving their resistance to axial forces (Park and Prausnitz, 2010). In case of the long needles group, no significant improvement in their mechanical strength was appreciated after increasing needle's width and so longer needles may need a thicker base to resist this force.

### 4.4 Insertion into Deka® film

**Fig. 6:**
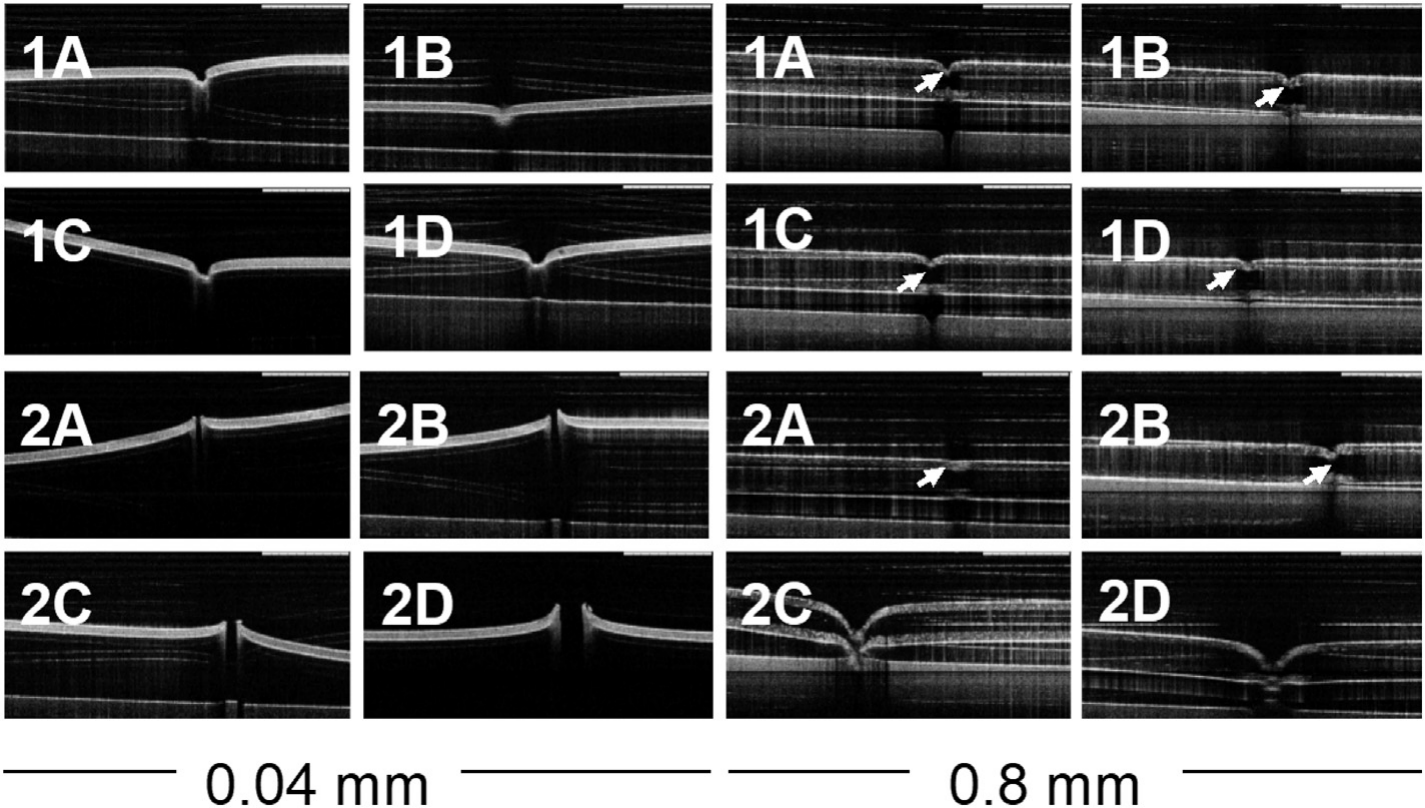
OCT images of Deka® film (0.04 and 0.8 mm thickness) after the insertion of single hydrogel-forming needles (scale bar represents 1 mm).

According to the German Institute for Standardisation and its guideline DIN 13097-4, hypodermic needles can be tested in terms of their insertion properties using a push-force-tester and poly(urethane) film, which emulates human skin (Deutsches Institut für Normung, 2009). Two films with different thicknesses (0.08 and 0.4 mm) are available on the market. Both films were used to test the insertion of single hydrogel-forming needles, using a Texture Analyser in compression mode and recording any change in the surface of films after the test with an OCT. Fig. 6 shows the results obtained for both groups of needles.

Needles with 2 mm lengths were not able to pierce the films. However, after the test, they were not broken or bent. In the case of needles of 4.5 mm, they pierced the thinner Deka® film but none of the prototypes was able to pierce the thicker one.

It can be seen in the images that the needles with a higher base width created a more noticeable indentation on the thicker film. Since both groups of needles did not experience any changes after the test, such as bending or breaking, this could suggest that they are sharp and strong enough to penetrate the films. However, length could be the factor that limits the penetration of films. Because of their elasticity, films tend to stretch when needles are pressed against them, hence, a longer needle could pierce films when they stop stretching.

### 4.5 Insertion into skin models

**Fig. 7:**
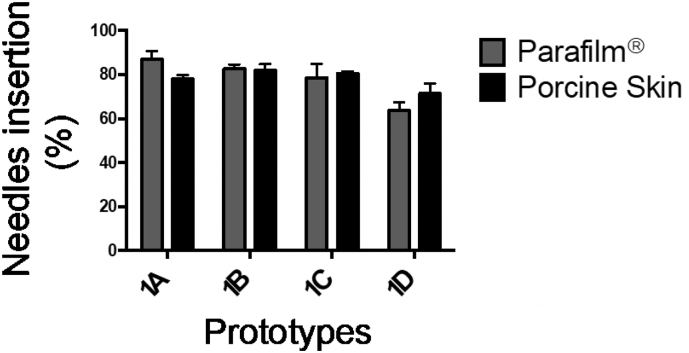
Insertion depth observed for short prototypes pressed for 30 s by a Texture Analyser in two skin models: Parafilm® (grey bars) and porcine skin (black bars) (mean + SD, n = 3).

Two different skin models were used to study the effect of needles' dimensions on their insertion properties: 20 or 40 consecutive layers of Parafilm® and porcine skin with underlying connective tissue. Fig. 7 shows the insertion depth of 2 mm long prototypes in both Parafilm® and porcine skin.

It can be clearly seen that all needles exhibited a similar percentage of height effectively inserted in each model, around 80% in all cases. When the Parafilm® group was assessed, only the prototype 1D showed a marked difference with the rest of the group, suggesting that an increased base width could be detrimental to the insertion process. However, this was not observed on porcine skin. Therefore, further studies with thicker needles would be necessary in order to draw more definitive conclusions.

**Fig. 8:**
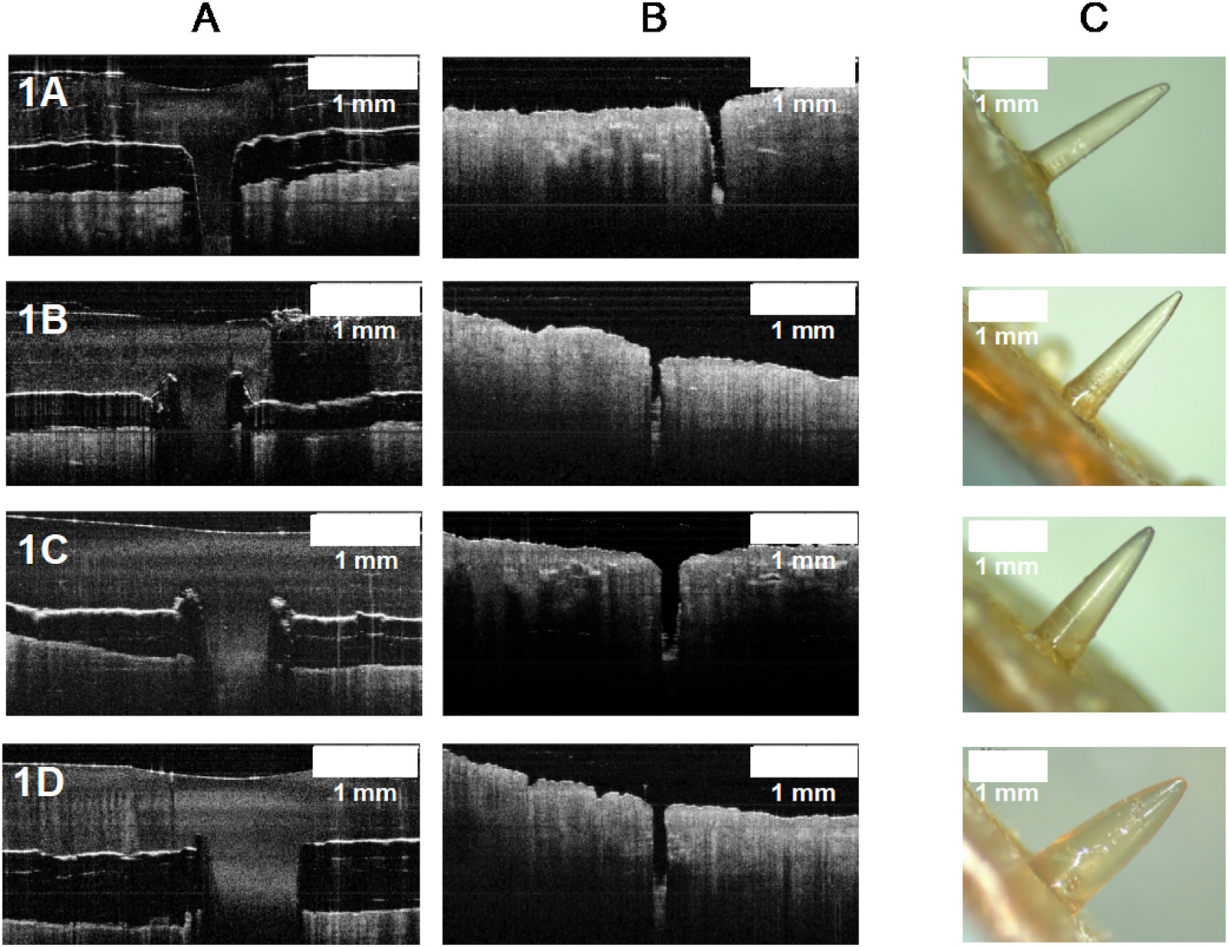
Images from insertion studies for short prototypes. A) OCT images of inserted needles; B) porcine skin after needle insertion and removal; C) needles after insertion and removal.

OCT was used to observe porcine skin during insertion and after removal of needles. Moreover, structural changes of needles after insertion and removal were evaluated using light microscopy. Representative images are shown in Fig. 8.

In all cases the skin was pierced, confirming the insertion of the needles and that there was not a significant effects in varying base width when porcine skin was used, as similar insertion values were observed (*p* < .05). In addition, it was confirmed that all needles of this group did not break or bend after insertion and removal. This could be mainly attributed to the low swelling properties of this formulation: although Gantrez® S-97 is a water-soluble copolymer, the formulation also includes a crosslinking agent, PEG with a low molecular weight. As previously reported, swelling properties of hydrogels made with Gantrez® S-97 are highly dependent on the molecular weight (MW) of the crosslinking agent used. Hydrogels crosslinked with a low molecular weight PEG showed rigid networks with high crosslink densities and, therefore, lower swelling rates (Raj Singh et al., 2009). Indeed, When PEG with a high molecular weight (10,000 Da) was used, swelling reached 250% whereas low molecular weight (200 Da), reached only 50% of swelling (Raj Singh et al., 2009).

**Fig. 9:**
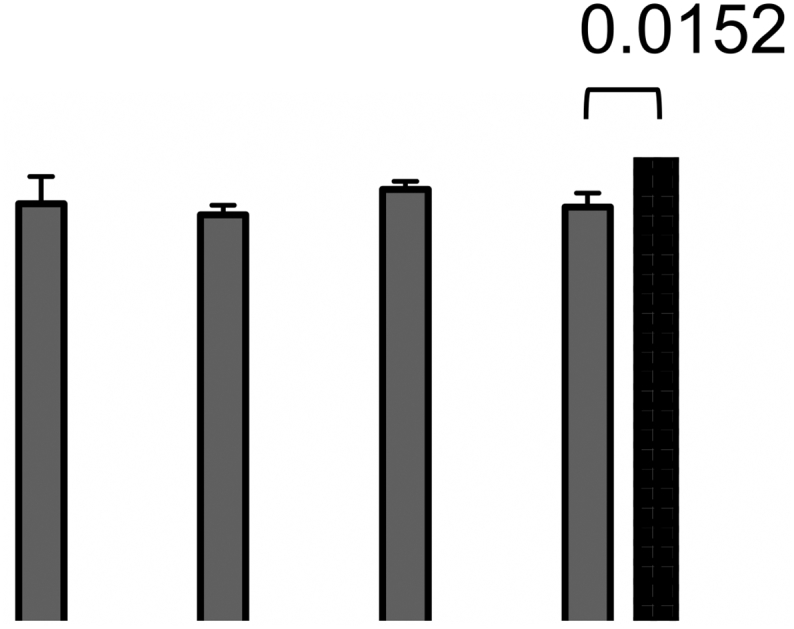
Insertion depth observed for long prototypes pressed for 30s by a Texture Analyser in two skin models: Parafilm® (grey bars) and porcine skin (black bars) (mean + SD, n = 3). △ represents prototypes that did not penetrate porcine skin. ▲ represents prototype that pierced the skin but bended.

In the case of longer needles, a clear effect of varying base width was observed between the two models, as it is shown in Fig. 9.

**Fig. 10:**
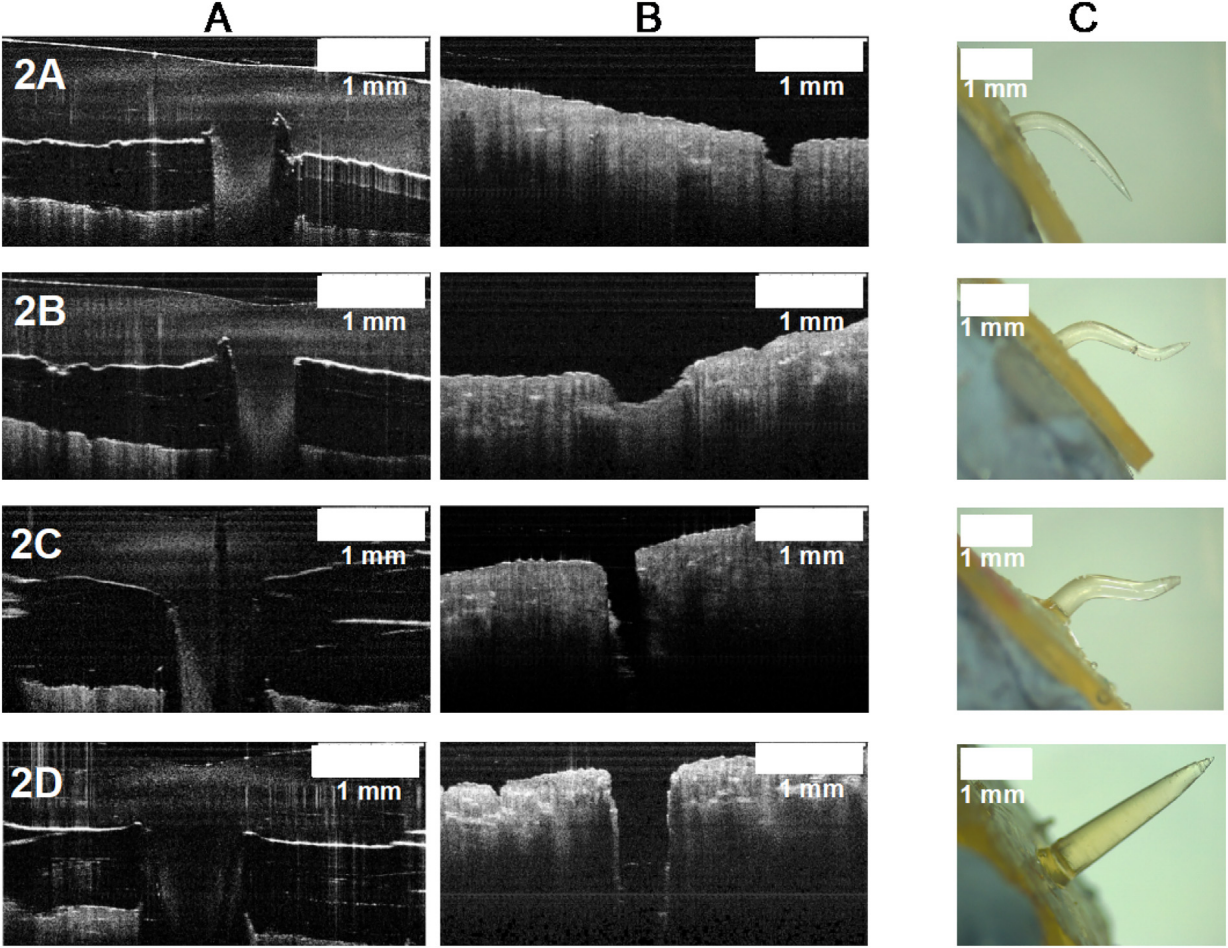
Images from insertion studies for long prototypes. A) OCT images of needles insertion; B) porcine skin after needle insertion and removal; C) needles after insertion and removal.

All the needles within this group were successfully inserted into Parafilm®, reaching similar percentages of insertion of at least 79%. An effect of varying needles' dimensions on their insertion was not appreciated as no differences where encountered when the group was compared. On the other hand, when studies were conducted using porcine skin, only the thicker needle (prototype 2D, 0.926 mm width) was successfully inserted. However, when insertion in Parafilm® and porcine skin for the prototype 2D was compared, statistical differences were encountered. OCT images were also obtained for this group to check the insertion process in porcine skin, as shown in Fig. 10.

OCT images showed an apparently effective insertion for all prototypes, however, by visualizing the pierced skin alone it was confirmed that needles were actually bending inside the skin. Prototypes 2C and 2D were capable of inserting and piercing the skin but the only needle that was removed intact after insertion was 2D, since 2C also bent but at a deeper zone of the skin.

It must be highlighted that 40 layers of Parafilm® were used to mimic the skin when testing longer needles, which was a very rigid structure in comparison to porcine skin. Hence, Parafilm® would not be a suitable model to evaluate longer needles as insertion profiles were not similar to those encountered with skin.

It has been previously shown that pain experienced by subjects following hypodermic needle puncture is significantly lower when needles of a smaller diameter are used (Edlich et al., 1987; Gill and Prausnitz, 2007; Wagø et al., 2016). Based on this criteria, prototype 1A was used for further studies. As only prototype 2D successfully inserted in porcine skin, despite the fact it has the thicker base in the case of longer needles, this one was used for further studies.

### 4.6 Coating of single needles with rhodamine B and characterisation

**Fig. 11:**
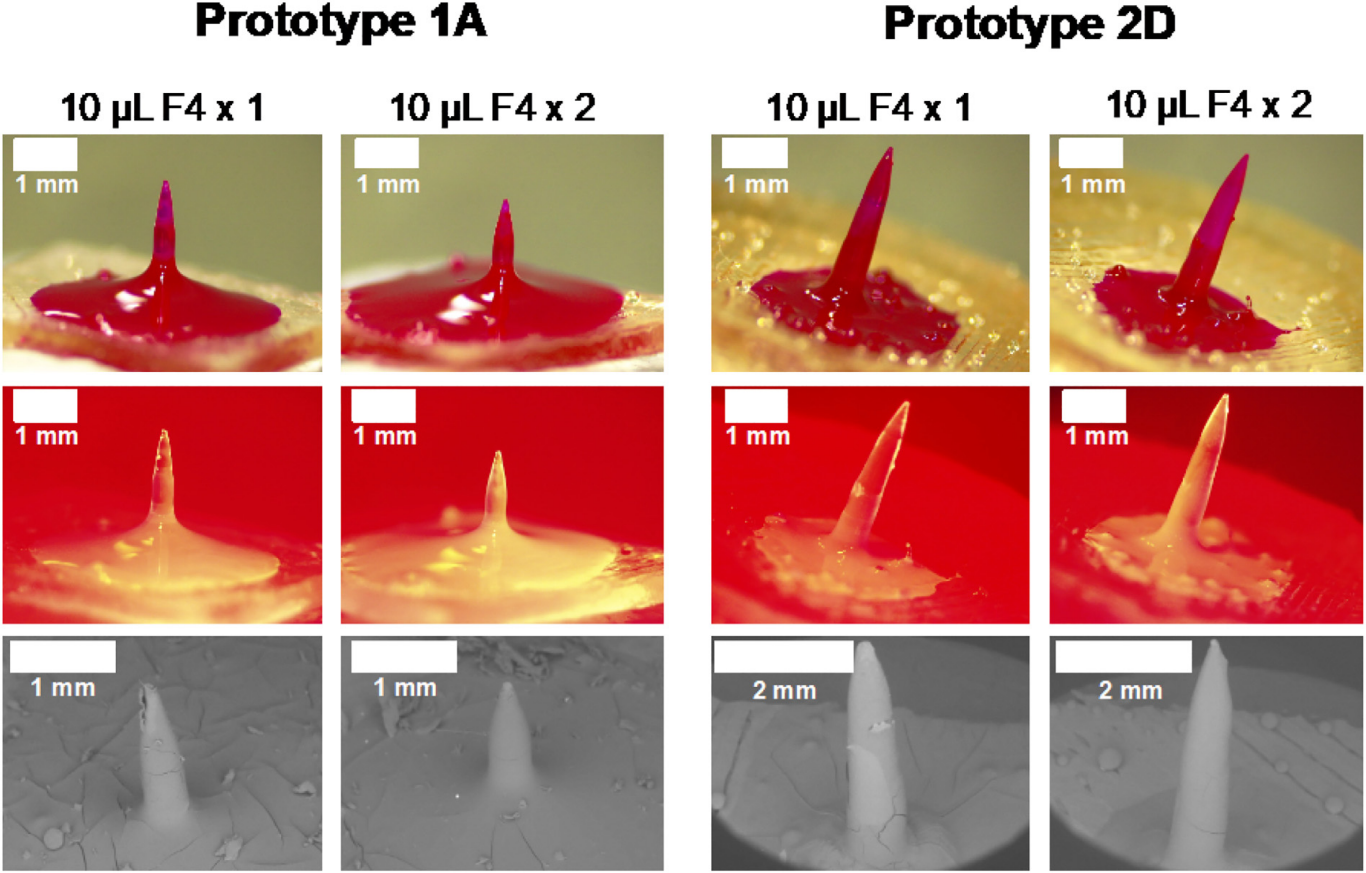
Coated hydrogel-forming needles, prototypes 1A and 2D, using 10 μL F4 (single and double coating). Representative images of light, fluorescent and scanning electron microscopy (scale bars represent 1 mm).

Several formulations were tested for the coating of prototypes 1A and 2D with rhodamine B, as model drug. PVP was used as film-forming agent, glycerol as plasticiser and Tween® 80 to decrease surface tension and allow a better distribution of the mixture across the needles and baseplate. Ethanol 99.5% was used as solvent since Gantrez® S-97 is poorly soluble on alcohols (Raj Singh et al., 2009), hence, there would be no changes to the needles' surface (such as swelling) during the coating/drying process. Film forming capacity of each formulation was initially tested by casting formulations on a siliconised release liner. Formulations F1, F2 and F3 turned into films that did not dry completely, which could be a consequence of the high content of glycerol and Tween® 80. Formulations F4 and F5 turned into dry films that could be removed from the siliconised paper with no changes. However, when the films were folded to evaluate their flexibility, F5 film broke immediately. Hence, formulation F4 was used to coat the needles. Needles were initially coated with 10 μL of formulation F4 and dried at room temperature overnight. Light, fluorescence and scanning electron microscopy (Fig. 11) showed that in both prototypes the distribution of rhodamine B was not uniform and some cracks were seen throughout the needle shaft, suggesting that the amount of coating was not enough. Hence, needles were coated twice, leaving one night of drying between coatings, using 10 μL of formulation F4 each time. In this case, needles were fully coated and fewer cracks were seen.

### 4.7 Rhodamine B penetration depth and quantification in porcine skin layers

**Fig. 12:**
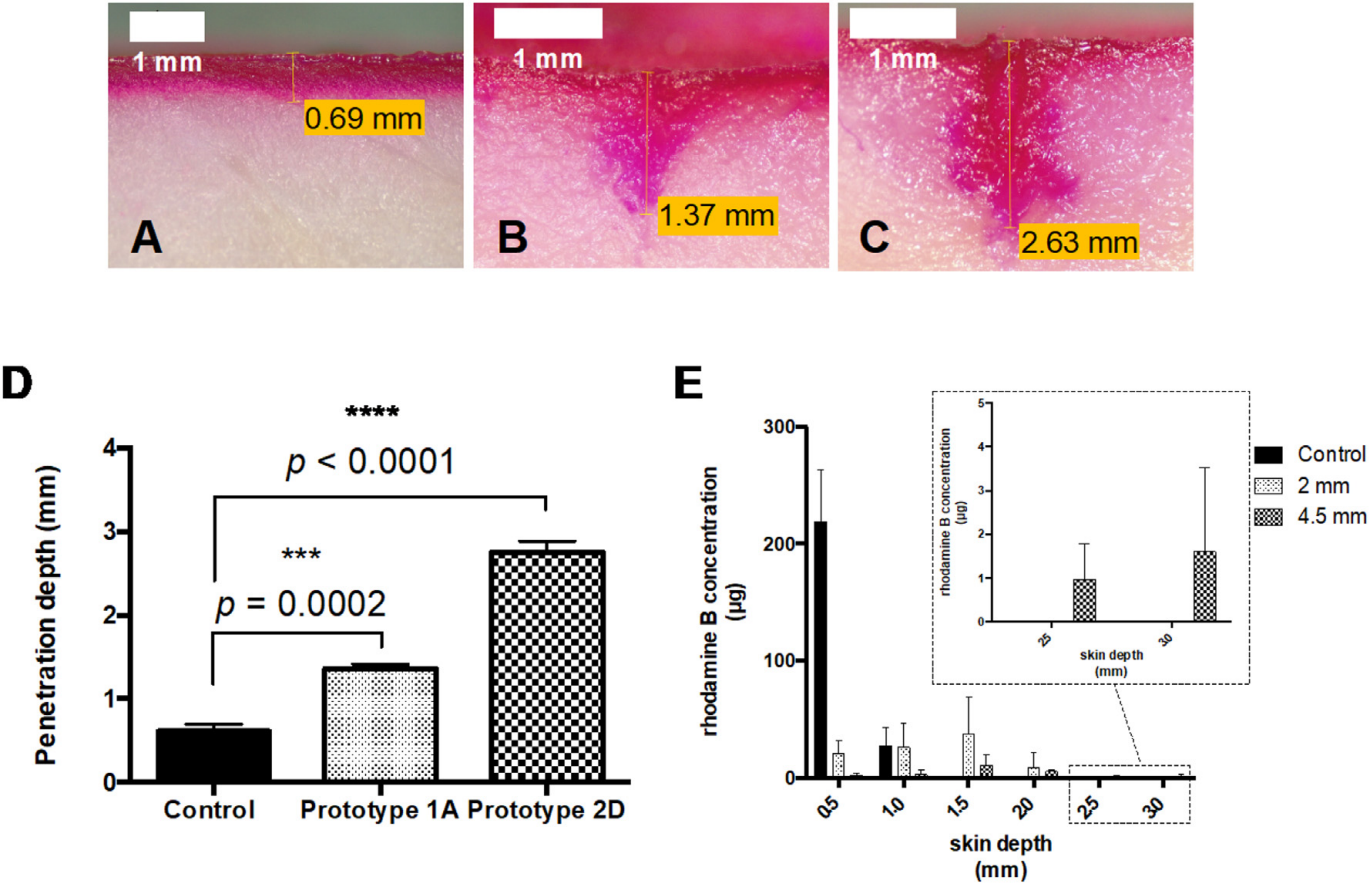
Penetration depth and quantification of rhodamine B on porcine skin. Representative images for penetration depth of A) control; B) prototype 1A; C) prototype 2D (scale bars represent 1 mm). D) Penetration depth of controls and single needles (mean ± SD, n = 3); E) Quantification of rhodamine B across the skin (mean ± SD, n = 3).

Coated needles were inserted into porcine skin, left for 15 min for the coating to dissolve and removed. Afterwards, pieces of skin were cut and the maximum penetration reached by rhodamine B was measured. A 2% w/w rhodamine B solution in ethanol 99.5% was used for control purposes. Representative images of rhodamine B penetration across porcine skin can be seen in Fig. 12.

When control was compared to short and long prototypes, significant differences were clearly seen as needles increased penetration depth of rhodamine B in 2 and 4.5 fold, respectively. Sections of porcine skin were obtained using a cryostat and model drug was quantified up to 3 mm depth (in intervals of 0.5 mm) using a validated HPLC method.

When a solution of rhodamine B (control) was placed on porcine skin, it was only detected up to 1.0 mm depth, being markedly more concentrated in the first 0.5 mm of skin. Single needle prototypes delivered rhodamine B notably deeper in the skin. Prototype 1A delivered around 40 μg between 1.0 and 1.5 mm and around 10 μg between 1.5 and 2.0 mm skin depth. Prototype 2D delivered around 3 μg between 2.0 and 3.0 mm of skin depth.

Coated needles used to study penetration depth of rhodamine B in porcine skin were removed intact after 15 min. To study if reinsertion of needles was possible, same prototypes were subjected to insertion again, pressing them manually into porcine skin for 30 s. Upon contact with the skin model, prototype 1A bended and 2D detached from the baseplate and broke (Supplemental material, Fig. S.2.), confirming that reinsertion of needles is not possible.

## 5 Conclusions

Several approaches were investigated in the manufacture of master moulds for the fabrication of single hydrogel-forming needles. When metallic needles were used as templates, fabrication of moulds was successfully achieved and a range of hydrogel-forming needles of defined lengths and base widths were manufactured in a reproducible way. A significant effect of needles' width at base on mechanical strength was appreciated on shorter prototypes, however, all of them inserted in both Parafilm® and porcine skin and were integrally removed. Longer prototypes were generally not inserted in porcine skin, except the thicker one, showcasing the effect of needles' width on insertion capability.

Double coating of needles with a mixture containing rhodamine B turned into fully coated needles, showing a homogenous distribution of the model drug throughout the needle's surface. In comparison to a control solution of rhodamine B, short needles increased the penetration depth of the model drug 2 times and in the case of long needles, 4.5 times. Similarly, quantification of rhodamine B on porcine skin showed that short single needles were able to deliver around 50 μg between 1.0 and 2.0 mm of skin depth. Long needles delivered a lower amount of drug in comparison to short needles, but reaching even deeper zones of the skin since around 5 μg where delivered between 2.0 and 3.0 mm depth.

These results show the feasibility of using a hydrogel for the preparation of needles and coating for deep intradermal delivery. The technology presented has the potential to overcome stability issues associated with liquid dosage forms required for conventional hypodermic needles or microinjection devices, as chemical interactions are less likely to take place in a dry state, avoiding the burden of cold chain. Furthermore, reinsertion of polymeric single needles is not possible, hence, they present enhanced safety in comparison to conventional hypodermic needles as needle-stick injuries and generation of hazardous waste can be significantly reduced.

Moreover, the combination of long needles and a coating formulation allows a rapid drug release into deep layers of the skin. Hence, quicker delivery rates could be achieved in comparison to conventional coated MNs arrays, where time for polymers dissolution or swelling and drug diffusion into the dermis is required. Altogether, these results confirm the potential of this innovative approach for rapid drug delivery below the epidermis. Inflammatory skin conditions (such as alopecia areata, psoriasis, keloid scars, scleroderma, among others), non-melanoma skin cancers and vaccination are examples of the wide range of biomedical areas where this technology could be applied.

## CRediT author statement

**Álvaro Cárcamo-Martínez**: Conceptualization, Methodology, Visualization, Investigation, Data curation, Writing - original draft. **Qonita Kurnia Anjani**: Methodology, Investigation, Validation. **Andi Dian Permana**: Investigation, Validation. **Ana Sara Cordeiro**: Conceptualization. **Eneko Larrañeta**: Writing - review & editing. **Ryan F. Donnelly**: Conceptualization, Methodology, Writing - review & editing, Supervision.

## Declaration of Competing Interest

The authors declare that they have no known competing financial interests or personal relationships that could have appeared to influence the work reported in this paper.

## Appendix A Supplementary data

Supplementary material Image 1
