# Supplementary material for: Coated polymeric needles for rapid and deep intradermal delivery
Source: Int J Pharm X. 2020 May 4;2:100048. doi: 10.1016/j.ijpx.2020.100048 (PMC7218294; doi:10.1016/j.ijpx.2020.100048)
Supplement: Supplementary file 1 — Supplementary material [file mmc1.docx]

Supplemental Material

**Coated polymeric needles for rapid and deep intradermal delivery**

Álvaro Cárcamo-Martínez^1^, Qonita Kurnia Anjani^1^, Andi Dian Permana^1,2^, Ana Sara Cordeiro^1^, Eneko Larrañeta^1^, Ryan F. Donnelly^1*^

*^1^School of Pharmacy, Queen’s University Belfast, Medical Biology Centre 97 Lisburn Road, Belfast BT9 7BL, UK*

*^2^Department of Pharmaceutics, Faculty of Pharmacy, Hasanuddin University, Makassar, Indonesia*

**Corresponding author:**

*School of Pharmacy, Queen’s University Belfast, 97 Lisburn Road, Belfast BT9 7BL, UK

[R.Donnelly@qub.ac.uk](mailto:R.Donnelly@qub.ac.uk)

**Keywords:** Polymeric needles, intradermal delivery, rhodamine B, optical coherence tomography.


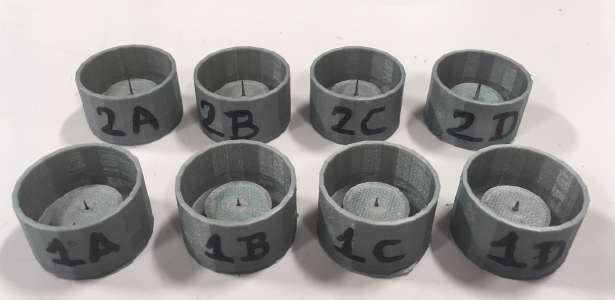


**Figure S.1.** Master moulds for the preparation of silicone moulds and single hydrogel-forming needles.

**Figure S.2.** Representative light microscopy images of coated prototypes 1A (A) and 2D mm (B) after trying reinserting them in porcine skin. Upon contact with porcine skin both needles broke and no insertion took place.
